# The Cellnovo Insulin Delivery System

**DOI:** 10.17925/EE.2017.13.01.13

**Published:** 2017-04-03

**Authors:** Stephen C Bain

**Affiliations:** Professor of Medicine (Diabetes), Swansea University, UK

**Keywords:** Diabetes, insulin, accuracy, continuous subcutaneous insulin infusion, patient

Autoimmune destruction of pancreatic beta-cells results in absolute insulin deficiency, type 1 diabetes mellitus (T1DM). Insulin therapy is required for people with T1DM to achieve an optimal glycated haemoglobin (HbA1c or A1c) along with controlled day-to-day blood glucose (BG) levels. While a variety of insulin delivery systems are available, factors such as the complexity of dosage calculation based on activity, diet and BG, the effectiveness of insulin delivery, ease of product use and ‘human’ issues, may affect concordance with treatment (see *Table 1*).^1,2^ Using an insulin delivery system which suits the patient’s lifestyle and addresses issues of delivery reliability may improve concordance and ameliorate BG variability. Continuous subcutaneous insulin infusion (CSII) therapy has been shown to:

- ensure accurate insulin delivery leading to improved A1c;^3^
- eliminate the unpredicatable effects of intermediate or long-acting insulin;^3^
- reduce severe low BG episodes; and^3^
- facilitate easier delivery of bolus insulin.^3^

In addition, a CSII system allows the patient flexibility in diet and meal times – users can exercise without having to eat large amounts of carbohydrate as the system can alter insulin delivery via a temporary basal rate.^3^

In this interview, Stephen C Bain describes the technical aspects of the Cellnovo mobile-connected diabetes management system.

## Can you tell us about the available technologies in diabetes management and the current unmet need?

As I have outlined in *Table 1*, concordance to therapy is dependent on a number of factors. For me, ease of use and reliability of dosage delivery are crucial. CSII pumps both deliver drug therapy and aid self-management, so patients using such technology should expect accurate and safe drug delivery and the facility to immediately identify when a problem has occurred. However, while many of the available durable and patch pumps have a programmable memory, safety lockout features and remote control functions,^4^ most ‘lock in’ this information, thus, by the time it is assessed by the patient and/or a clinician, much of its value and relevance is lost.

## What makes the Cellnovo system different to other insulin delivery devices?

It is different because it is the first mobile diabetes management system, providing intuitive operation, internet connectivity and real-time tracking. The system comprises three core parts:

- a slim insulin pump with in-built activity monitor, which wirelessly connects to the app-based touch-screen handset;
- an app-based touch-screen handset; this features an integral BG monitor and a mobile data connection; the handset automatically records insulin dose, exercise and BG while also allowing for storage of diet information; and
- a comprehensive and secure web-based clinical management tool.

**Table 1:** Factors influencing effectiveness of regimens and/or concordance^1,2^

**Care-related:** A lack of a team approach to care^1^ Reluctance to commence therapy by patients and clinicians^1^ A negative impact on relationship with family/friends, physical health^2^ Low person-centred chronic illness care and support^2^ Poor participation in and/or lack of available diabetes educational programmes/activities to help manage their diabetes^2^
**Patient-related:** Low adherence to diet, medication/testing regimens and appointment keeping Diabetes-related stress (feelings of shock, anxiety, helplessness, fear of complications, social burden)^2^ Psychological issues interfering with concordance^2^ Depression^2^ ‘Poor’ or ‘very poor’ quality of life^2^ Diabetes-related distress^2^ Medication interfering with normal life^2^

The system was created to reduce adverse incidents through its safety features and its ability to capture real-time data for rapid analysis and intervention by the patient and/or clinician where necessary.

## What safety features does the Cellnovo device have?

The system has a number of alarms which relate to insulin delivery, occlusion, cartridge expiry alarm and temperature risk; the latter alarm alerts the patient if the ambient temperature exceeds 37 °C for a period of time, (i.e., the threshold where insulin is known to start to break down and become less effective). Box 1 outlines all the safety and function features.

## Could you describe the mechanism of action of the miniaturised pump and how it differs from other pumps?

Most standard insulin pumps are simply a battery-driven automated syringe. In other pumps, this type of system is commonly used and comprises a modified injection syringe/cartridge filled with rapid-acting insulin, which is placed inside the pump. The cartridge is then emptied by means of a plunger, which advances by rotating along a helical thread. Insulin is released at variable speeds through a catheter into a subcutaneous tissue.

The Cellnovo pump is different from these other types of pumps in that it comprises two elements: a durable component and a disposable component.

The disposable component (the insulin cartridge) contains:

- the micro-actuator;
- the micro-valving system;
- an occlusion sensor; and
- an individual identification (ID) chip.

**Figure 1:**
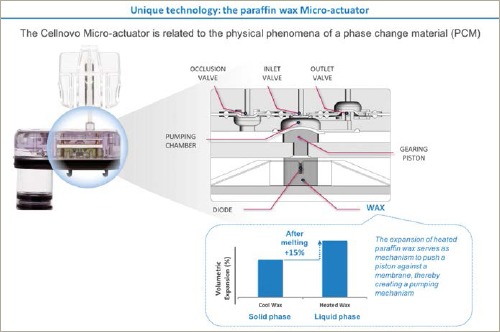
Micro-actuator

Let me explain how these components work. In the micro-actuator, wax is heated, causing it to expand; this forces the piston to move, displacing a small amount of insulin from the pumping chamber, out of the insulin cartridge, through the infusion set and into the patient (see *Figure 1*). Over time, doses deliver insulin as quasi-continuous basal delivery or as a larger bolus dose. A basal rate of 1 unit/hour would consist of 0.05 unit doses – a dose occurs every three minutes (20 doses in 1 hour). For a bolus dose, a mix of 0.1 and 0.05 units is used, due to the relatively large number of doses that need to be delivered in a short period of time. The 0.1 unit doses are used predominantly and 0.05 unit doses are used at the end of delivery to ensure accuracy.

The temperature of the wax in a solid state is measured in order to determine the correct activation time for the heating phase of the actuation cycle. In simple terms, the colder the wax, the more energy is needed to elevate the temperature to its liquid transition temperature. After the wax is heated and the first dose is delivered, the wax will cool and contract, returning to its solid state and volume.

The size of the basal rate will determine when the next dose is required; for example, larger basal rates have more doses to deliver per hour so the period between delivery doses will be shorter. The next heating cycle will begin at this point, again taking a measurement of the relative temperature of the wax in order to determine the activation time. This pulsing provides better control of accuracy of delivery per dose compared to some other commercially available patch pumps due to the feedback mechanisms described later.^5^

## How is insulin flow controlled within the pump?

Via the patented micro-fluidic system, which contains two one-way valves (Patent No. GB2443260). A valve will remain closed until enough pressure is exerted on it, causing it to open and allowing forward flow. The inlet valve is located at the entry to the pumping chamber (exiting from the reservoir), the outlet valve on the exit. Movement of the piston creates a rise in pressure within the pumping chamber, which is much greater than that which the outlet valve can withstand, thus opening the valve and displacing a volume of insulin out of the insulin cartridge to the patient. The one-way valve system prevents back-flow of insulin, so it is not susceptible to either the siphoning effects that can be seen in other insulin pumps,^6^ nor to changes in external pressures, for example, at high altitudes during aircraft flights or skiing.

## How is insulin delivery monitored?

**Figure 2:**
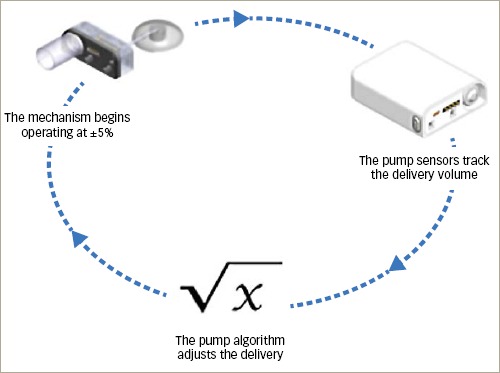
Intelligent Delivery System

Through the Intelligent Delivery System (IDS), which comprises a sensor coupled with unique software algorithms (see *Figure 2*). The patented technology (GB2464114) continuously monitors insulin delivered by measuring the amount of insulin in the reservoir and correcting the delivery rate as required, thus ensuring consistent and accurate delivery. In addition, the system can take immediate action in the event that it is unable to correct an insulin delivery error; for example, if a fault is detected, the system can cease pumping, disconnect the insulin cartridge from the pump using the automatic release mechanism and notify the patient with an alarm. Thus, there is minimal risk of the patient receiving unintended insulin delivery. Once disconnected, the insulin cartridge cannot be reinserted, so a new cartridge must be used.

The core technology within the IDS is the delivery sensor (DS). This comprises a spring-loaded sensor which sits against the plunger within the insulin cartridge’s reservoir and applies a constant pressure to the insulin volume. The sensor is able to detect its physical position within the reservoir and then this position is translated into a measurement that equates to the volume of insulin that is present. Therefore, the DS detects whether or not the reservoir level is decreasing at the correct rate of delivery.

The DS is accurate to 0.001U and is calibrated during manufacture to ensure insulin delivery is consistent and accurate from pump to pump. If the DS exhibits a fault, then the insulin cartridge will be immediately disconnected from the pump and the patient will be notified by an alarm. The cartridge cannot be re-inserted once it has been disconnected due to the system remembering the state of the disconnected cartridge.

## How is the bolus size determined?

The IDS software algorithms ensure that the actual basal/bolus delivered matches the programmed rate. In terms of basal rate, the pump samples the reservoir using the DS and measures the insulin volume after a pre-determined number of doses have been delivered. The software algorithm calculates an average dose size and then determines whether or not the average dose size is within acceptable tolerances to the nominal dose size of 0.05 units. If the average dose size is outside of the acceptable tolerances of ±5% then the software will adjust the activation time to compensate.

The system also monitors bolus delivery, ensuring accuracy through intermittent checks and halting delivery once the programmed dose has been delivered.

The touch-screen, app-based handset contains all of the pump controls, a built-in BG meter, food library, activity apps and an automated journal. The system’s technology can provide bolus recommendations for delivery based on patient-programmed inputs such as carbohydrate amount, insulin to carbohydrate ratio, BG correction ratio, time to target (the time it takes the BG level to reach the mid-point of the target range after a bolus), and the desired BG target range.

## How are data stored and accessed by the patient?

Data are stored in a secure web-based management system, ‘Cellnovo Online’, which can be accessed at any time by the patient via the ‘Members’ Portal’. Here the patient can access analytical tools and graphs, which allow them to review their pump data in order to facilitate optimal diabetes control. Cellnovo Online also provides an extensive suite of support tools such as the Cellnovo Academy, Knowledge-Based Articles (KBA), FAQs, iChat and User Guides.

The ‘Clinicians’ Portal’ allows healthcare professionals to view the data for their patients, and to access a similar set of analytical tools, facilitating more effective support to those who need it.

## Should the patient attend a clinic to discuss the data?

As data are achieved in real time and are immediately available over a secure mobile web connection, potential clinical or user problems that might otherwise go unnoticed can be seen by the healthcare team for immediate attention if necessary. Conversely, patients who are managing well will no longer need to attend a clinic just to be informed that their diabetes management is on target. Clinic visits can be focused on interpreting and responding to information instead of time being spent piecing it together from memories and hand-written journals, and no need for downloading of data on the day of the clinic visit. With access to all the data, patients and clinicians are able to make swift assessments and decisions with the confidence of accurate and comprehensive knowledge.

## Is there anything else you would like to say about the system?

The culmination of all of these features with particular respect to the online connectivity and the ID Chip (which is used to register each insulin cartridge), allows for remote investigation of product performance, postmarket surveillance and vigilance activities. In partnership with patients, professionals are able to diagnose product queries and complaints, and often solve any use issues with the capability to make data available to regulators. Additionally, the full traceability of the product from manufacture to disposal can ensure that batch issues are quickly identified and allows for automatic replacement of supplies.

## Which patients are likely to benefit most from this technology?

The system plays to the particular requirements of younger T1DM patients, as they are generally more ‘tech-savvy’ and active, but this is not to exclude other age groups; quality of life is improved using a CSII system.^7^ Outcomes in clinical indicators and quality of life indicators also suggest that such systems are preferable to multiple daily injection regimens.^8^

## Does the system have any drawbacks?

Interestingly, anecdotal feedback from users indicates that the safety alarms, one of the main tenets of the system, can be embarrassing if they sound in public. The fact that the insulin cartridge automatically disconnects after 72 hours can be inconvenient if it occurs during the night, or if the patient is not at home, or they do not have a new cartridge to hand. Further development of the system, for example, by widening appropriate alarm parameters, should balance safe and accurate delivery of insulin and facilitate a ‘normal’ life for the patient.

Some users initially felt they had to be more ‘tech-savvy’ to understand how to use the handset and online interface, but over time they have become more competent. Others have expressed concern that their data are transmitted to an online portal, but have since conveyed satisfaction in regards to the security and completeness of the information, as well as the ability to share their data with their clinical team.

## Conclusion

This novel system facilitates self-management. The safety features plus the programmable handset facilitate safe and effective insulin delivery.

**Box 1: Cellnovo features**

- Cellnovo Intelligent Delivery System – unique Cellnovo technology which monitors insulin delivery accuracy and can correct for insulin delivery errors (within operating tolerances). If the delivery system cannot correct the delivery error, then it will notify the patient in the form of an alarm and may take immediate action in some situations in order to maintain the patient’s safety. It allows a 24-hour Basal Profile, programmable in hourly increments, a Maximum Basal and Bolus Rate setting to limit the maximum programmable rates, and cannula prime to ensure the infusion set cannula is filled with insulin once the infusion pump and set are attached to the body.
- Cellnovo Bolus Calculator – unique Cellnovo technology that can provide bolus recommendations for delivery based on patient-programmed inputs such as carbohydrates, insulin to carbohydrate ratio, BG correction ratio, time to target and desired BG target range.
- Automatic release mechanism – a mechanism which automatically disconnects the insulin cartridge in over-delivery situations or when the insulin delivery does not match the programmed rate.
- Identification chip (ID Chip) – a microchip which is unique to each Insulin Cartridge which prevents re-use of a faulty Insulin Cartridge or use of a non-Cellnovo Insulin Cartridge. The ID Chip also registers the use of the individual insulin cartridge with the Cellnovo backend systems allowing for tracing use that can be utilised in post market surveillance and vigilance.
- Delivery Sensor – patented Cellnovo technology (GB2464114) which measures physical insulin delivery.
- Insulin delivery alarms – a set of alarms that monitor for delivery errors and alarms in cases of unintended delivery, under-delivery or no delivery in both basal and bolus delivery.
- Occlusion alarm – an alarm that monitors for blockages in the infusion set. Set at below 1 unit of missed insulin.
- Temperature risk alarm – ambient temperature is monitored and alerts the patient if this exceeds 37 °C for a period of time, (i.e. the threshold where insulin is known to start to break down and become less effective).
- Cartridge Expiry alarm – an alarm that triggers at 72-hours usage of the cartridge, to signify that the insulin effectiveness cannot be guaranteed beyond 72 hours. The cartridge cannot be used passed this time.
- Handset screen lock – minimises the chances of unintended access to the handset’s touch screen.
- Hold to Confirm – a double confirmation feature which minimises the chances of inadvertent changes to insulin therapy.
- Encryption – secure communication which prevents unauthorised third party access to the patient’s pump and a further security feature which ensures that all data transferred or stored in Cellnovo Online cannot be altered, lost or accessed by unauthorised third parties.
- User Registration – a mandatory process required to enable the handset for first time use and allows transfer and storage of patient data to Cellnovo Online. Log-in Authentication prevents unauthorised third party access to patient data through automated means.
- Data Integrity Verification – a standard security feature which checks that all patient data in Cellnovo Online matches the data in each patient’s handset during transmission, storage and retrieval activities.
